# [μ-Bis(diphenyl­phosphan­yl)ethane-1:2κ^2^ *P*:*P*′]nona­carbonyl-1κ^3^ *C*,2κ^3^ *C*,3κ^3^ *C*-[tris­(4-(meth­oxy­phen­yl)arsane-3κ*As*]-*triangulo*-triruthenium(0) chloro­form monosolvate

**DOI:** 10.1107/S160053681100047X

**Published:** 2011-01-15

**Authors:** Omar bin Shawkataly, Imthyaz Ahmed Khan, Siti Syaida Sirat, Chin Sing Yeap, Hoong-Kun Fun

**Affiliations:** a Chemical Sciences Programme, School of Distance Education, Universiti Sains Malaysia, 11800 USM, Penang, Malaysia; b X-ray Crystallography Unit, School of Physics, Universiti Sains Malaysia, 11800 USM, Penang, Malaysia

## Abstract

The asymmetric unit of the title *triangulo*-triruthenium compound, [Ru_3_(C_21_H_21_AsO_3_)(C_26_H_24_P_2_)(CO)_9_]·CHCl_3_, consists of one mol­ecule of the *triangulo*-triruthenium complex and one chloro­form solvent mol­ecule. The bis(diphenyl­phosphan­yl)ethane ligand bridges an Ru—Ru bond and the monodentate arsane ligand bonds to the third Ru atom. Both the arsane and phosphine ligands are equatorial with respect to the Ru_3_ triangle. Additionally, each Ru atom carries one equatorial and two axial terminal carbonyl ligands. The three arsane-substituted benzene rings make dihedral angles of 52.72 (19), 63.03 (19) and 88.19 (19)° with each other. The dihedral angles between the two benzene rings are 85.8 (2) and 89.2 (2)° for the two diphenyl­phosphanyl groups. In the crystal, mol­ecules are linked together into a three-dimensional network *via* inter­molecular C—H⋯O hydrogen bonds. Weak inter­molecular C—H⋯π inter­actions further stabilize the crystal structure.

## Related literature

For general background to *triangulo*-triruthenium derivatives, see: Bruce *et al.* (1985 ▶, 1988*a* ▶,*b* ▶)1985 ▶,. For related structures, see: Shawkataly *et al.* (1998 ▶, 2004 ▶, 2010*a* ▶,*b* ▶). For the synthesis of Ru_3_(CO)_10_(μ-Ph_2_PCH_2_CH_2_PPh_2_), see: Bruce *et al.* (1983 ▶). For the stability of the temperature controller used in the data collection, see: Cosier & Glazer (1986 ▶).
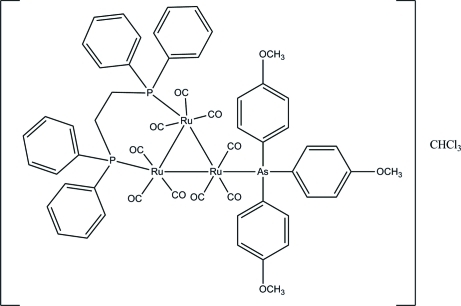


## Experimental

### 

#### Crystal data

[Ru_3_(C_21_H_21_AsO_3_)(C_26_H_24_P_2_)(CO)_9_]·CHCl_3_
*M* *_r_* = 1469.36
Monoclinic, 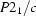

*a* = 13.1097 (2) Å
*b* = 19.8034 (4) Å
*c* = 22.5603 (4) Å
β = 98.689 (1)°
*V* = 5789.81 (18) Å^3^
*Z* = 4
Mo *K*α radiation
μ = 1.59 mm^−1^
*T* = 100 K
0.58 × 0.26 × 0.04 mm

#### Data collection

Bruker SMART APEXII CCD area-detector diffractometer
Absorption correction: multi-scan (*SADABS*; Bruker, 2009 ▶) *T*_min_ = 0.458, *T*_max_ = 0.946
64045 measured reflections
16965 independent reflections
12581 reflections with *I* > 2σ(*I*)
*R*_int_ = 0.051

#### Refinement

*R*[*F* ^2^ > 2σ(*F* ^2^)] = 0.067
*wR*(*F* ^2^) = 0.108
*S* = 1.11
16965 reflections
706 parameters
H-atom parameters constrained
Δρ_max_ = 2.13 e Å^−3^
Δρ_min_ = −1.16 e Å^−3^

### 

Data collection: *APEX2* (Bruker, 2009 ▶); cell refinement: *SAINT* (Bruker, 2009 ▶); data reduction: *SAINT*; program(s) used to solve structure: *SHELXTL* (Sheldrick, 2008 ▶); program(s) used to refine structure: *SHELXTL*; molecular graphics: *SHELXTL*; software used to prepare material for publication: *SHELXTL* and *PLATON* (Spek, 2009 ▶).

## Supplementary Material

Crystal structure: contains datablocks global, I. DOI: 10.1107/S160053681100047X/sj5086sup1.cif

Structure factors: contains datablocks I. DOI: 10.1107/S160053681100047X/sj5086Isup2.hkl

Additional supplementary materials:  crystallographic information; 3D view; checkCIF report

## Figures and Tables

**Table 1:** Hydrogen-bond geometry (Å, °) *Cg*1, *Cg*2 and *Cg*3 are the centroids of the C34–C39, C7–C12 and C1–C6 benzene rings, respectively.

*D*—H⋯*A*	*D*—H	H⋯*A*	*D*⋯*A*	*D*—H⋯*A*
C19—H19*A*⋯O10^i^	0.93	2.34	3.269 (5)	176
C43—H43*A*⋯O10^ii^	0.93	2.52	3.065 (5)	118
C47—H47*C*⋯O1^iii^	0.96	2.56	3.400 (6)	146
C13—H13*A*⋯*Cg*1^iv^	0.97	2.96	3.856 (4)	155
C23—H23*A*⋯*Cg*2^v^	0.93	2.73	3.467 (4)	137
C46—H46*A*⋯*Cg*3^iii^	0.93	2.83	3.623 (4)	145

Symmetry codes: (i) 
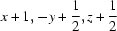
; (ii) 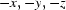; (iii) 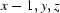; (iv) 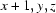; (v) 
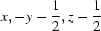
.

**Fig. 1.**
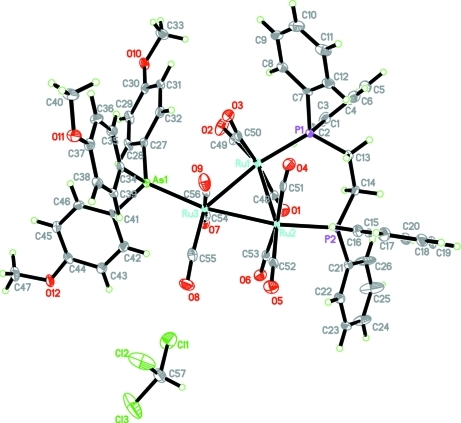
The molecular structure of the title compound with 50% probability ellipsoids for non-H atoms.

**Fig. 2.**
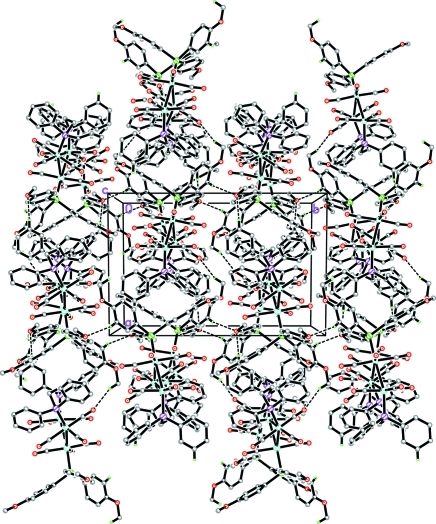
The crystal packing of the title compound, viewed down the c axis, showing the molecules linked into a three-dimensional network. Hydrogen atoms that are not involved in the hydrogen-bonding (dashed lines) and solvent molecules have been omitted for clarity.

## supplementary crystallographic information

### Comment

A large number of substituted derivatives of the type Ru_3_(CO)_12-n_*L*_n_ (*L* = group 15 ligand) have been reported (Bruce *et al.*, 1985, 1988*a*,*b*). As part of our study on the substitution of transition metal-carbonyl clusters with mixed-ligand complexes, we have published several structures of *triangulo*-triruthenium-carbonyl clusters containing mixed P/As and P/Sb ligands (Shawkataly *et al.*, 1998, 2004, 2010*a*,*b*). Herein we report the synthesis and structure of the title compound.

The asymmetric unit of the title compound consists of one molecule of the *triangulo*-triruthenium complex and one molecule of chloroform (Fig. 1). The bis(diphenylphosphanyl)ethane ligand bridges the Ru1–Ru2 bond and the monodentate arsane ligand bonds to the Ru3 atom. Both the arsane and phosphine ligands are equatorial with respect to the Ru_3_ triangle. Additionally, each Ru atom carries one equatorial and two axial terminal carbonyl ligands. The three arsane-substituted benzene rings make dihedral angles (C27–C32/C33–C38, C27–C32/C39–C44 and C33–C38/C39–C44) of 52.72 (19), 63.03 (19) and 88.19 (19)° with each other respectively. The dihedral angles between the two benzene rings (C1–C6/C7–C12 and C15–C20/C21–C26) are 85.8 (2) and 89.2 (2)° for the two diphenylphosphanyl groups respectively. The three methoxyphenyl groups are planar with torsion angles C33–O10–C30–C31 = 5.6 (6)°, C40–O11–C37–C36 = -8.9 (6)° and C47–O12–C44–C45 = -6.5 (6)°.

In the crystal packing, the molecules are linked together *via* intermolecular C19—H19A···O10, C43—H43A···O10 and C47—H47C···O1 hydrogen bonds (Table 1) into a three-dimensional nextwork (Fig. 2). Weak intermolecular C—H···π interactions (Table 1) further stabilize the crystal structure.

### Experimental

All manipulations were performed under a dry oxygen-free nitrogen atmosphere using standard Schlenk techniques. All solvents were dried over sodium and distilled from sodium benzophenone ketyl under dry oxygen free nitrogen. Tris(4-methoxyphenyl)arsane is prepared by the reaction of AsCl_3_ with 4-OCH_3_C_6_H_4_MgBr and Ru_3_(CO)_10_(µ-Ph_2_PCH_2_ CH_2_PPh_2_) was prepared by reported procedure (Bruce *et al.*,1983). The title compound was obtained by refluxing equimolar quantities of Ru_3_(CO)_10_(µ-Ph_2_PCH_2_CH_2_PPh_2_) and tris(4-methoxylphenyl)arsane in hexane under nitrogen atmosphere. Crystals suitable for X-ray diffraction were grown by slow solvent / solvent diffusion of CH_3_OH into CHCl_3_.

### Refinement

All hydrogen atoms were positioned geometrically and refined using a riding model with C—H = 0.93–0.97 Å and *U*_iso_(H) = 1.2 or 1.5 *U*_eq_(C). A rotating group model was applied for the methyl groups. The maximum and minimum residual electron density peaks of 2.13 and -1.16 e Å^-3^ were located 0.85 Å and 1.41 Å, respectively, from the RU3 atom.

### Crystal data

**Table:**

[Ru_3_(C_21_H_21_AsO_3_)(C_26_H_24_P_2_)(CO)_9_]·CHCl_3_	*F*(000) = 2920
*M**_r_* = 1469.36	*D*_x_ = 1.686 Mg m^−^^3^
Monoclinic, *P*2_1_/*c*	Mo *K*α radiation, λ = 0.71073 Å
Hall symbol: -P 2ybc	Cell parameters from 9908 reflections
*a* = 13.1097 (2) Å	θ = 2.2–30.0°
*b* = 19.8034 (4) Å	µ = 1.59 mm^−^^1^
*c* = 22.5603 (4) Å	*T* = 100 K
β = 98.689 (1)°	Plate, brown
*V* = 5789.81 (18) Å^3^	0.58 × 0.26 × 0.04 mm
*Z* = 4	

### Data collection

**Table:**

Bruker SMART APEXII CCD area-detector diffractometer	16965 independent reflections
Radiation source: fine-focus sealed tube	12581 reflections with *I* > 2σ(*I*)
graphite	*R*_int_ = 0.051
φ and ω scans	θ_max_ = 30.1°, θ_min_ = 1.9°
Absorption correction: multi-scan (*SADABS*; Bruker, 2009)	*h* = −18→14
*T*_min_ = 0.458, *T*_max_ = 0.946	*k* = −23→27
64045 measured reflections	*l* = −31→31

### Refinement

**Table:**

Refinement on *F*^2^	Primary atom site location: structure-invariant direct methods
Least-squares matrix: full	Secondary atom site location: difference Fourier map
*R*[*F*^2^ > 2σ(*F*^2^)] = 0.067	Hydrogen site location: inferred from neighbouring sites
*wR*(*F*^2^) = 0.108	H-atom parameters constrained
*S* = 1.11	*w* = 1/[σ^2^(*F*_o_^2^) + (0.0263*P*)^2^ + 20.0015*P*] where *P* = (*F*_o_^2^ + 2*F*_c_^2^)/3
16965 reflections	(Δ/σ)_max_ < 0.001
706 parameters	Δρ_max_ = 2.13 e Å^−^^3^
0 restraints	Δρ_min_ = −1.16 e Å^−^^3^

### Special details

**Table:**

Experimental. The crystal was placed in the cold stream of an Oxford Cryosystems Cobra open-flow nitrogen cryostat (Cosier & Glazer, 1986) operating at 100.0 (1) K.
Geometry. All e.s.d.'s (except the e.s.d. in the dihedral angle between two l.s. planes) are estimated using the full covariance matrix. The cell e.s.d.'s are taken into account individually in the estimation of e.s.d.'s in distances, angles and torsion angles; correlations between e.s.d.'s in cell parameters are only used when they are defined by crystal symmetry. An approximate (isotropic) treatment of cell e.s.d.'s is used for estimating e.s.d.'s involving l.s. planes.
Refinement. Refinement of *F*^2^ against ALL reflections. The weighted *R*-factor *wR* and goodness of fit *S* are based on *F*^2^, conventional *R*-factors *R* are based on *F*, with *F* set to zero for negative *F*^2^. The threshold expression of *F*^2^ > σ(*F*^2^) is used only for calculating *R*-factors(gt) *etc*. and is not relevant to the choice of reflections for refinement. *R*-factors based on *F*^2^ are statistically about twice as large as those based on *F*, and *R*- factors based on ALL data will be even larger.

### Fractional atomic coordinates and isotropic or equivalent isotropic displacement parameters (Å^2^)

**Table:**

	*x*	*y*	*z*	*U*_iso_*/*U*_eq_	
Ru1	0.32826 (2)	0.210241 (17)	0.048812 (14)	0.01175 (8)	
Ru2	0.36871 (2)	0.252908 (17)	0.170465 (14)	0.01048 (7)	
Ru3	0.16553 (2)	0.216924 (17)	0.120575 (14)	0.01162 (8)	
As1	−0.00362 (3)	0.19620 (2)	0.060148 (19)	0.01228 (9)	
P1	0.47394 (8)	0.25345 (6)	0.01451 (5)	0.0132 (2)	
P2	0.54788 (8)	0.25502 (6)	0.18788 (5)	0.0119 (2)	
O1	0.4486 (2)	0.08901 (16)	0.10796 (14)	0.0228 (7)	
O2	0.2482 (3)	0.11519 (19)	−0.05325 (16)	0.0355 (9)	
O3	0.2033 (2)	0.33319 (16)	−0.00532 (14)	0.0207 (7)	
O4	0.3584 (2)	0.38885 (16)	0.10400 (14)	0.0220 (7)	
O5	0.3269 (3)	0.31436 (17)	0.28767 (14)	0.0257 (8)	
O6	0.3642 (2)	0.11144 (16)	0.22650 (14)	0.0220 (7)	
O7	0.2049 (2)	0.06503 (16)	0.10891 (14)	0.0227 (7)	
O8	0.1159 (3)	0.20591 (18)	0.24703 (14)	0.0290 (8)	
O9	0.1242 (2)	0.37007 (17)	0.11541 (16)	0.0258 (8)	
O10	0.0087 (2)	0.09243 (16)	−0.19322 (13)	0.0188 (7)	
O11	−0.2495 (2)	0.45858 (16)	0.04417 (14)	0.0216 (7)	
O12	−0.2560 (2)	−0.01650 (16)	0.16866 (14)	0.0203 (7)	
C1	0.5493 (3)	0.1957 (2)	−0.02549 (18)	0.0161 (9)	
C2	0.5315 (3)	0.1269 (2)	−0.0287 (2)	0.0204 (10)	
H2A	0.4790	0.1087	−0.0102	0.025*	
C3	0.5906 (4)	0.0844 (3)	−0.0590 (2)	0.0268 (11)	
H3A	0.5781	0.0381	−0.0601	0.032*	
C4	0.6673 (4)	0.1106 (3)	−0.0874 (2)	0.0267 (12)	
H4A	0.7065	0.0822	−0.1079	0.032*	
C5	0.6861 (4)	0.1789 (3)	−0.0855 (2)	0.0303 (12)	
H5A	0.7378	0.1965	−0.1050	0.036*	
C6	0.6287 (4)	0.2218 (3)	−0.0549 (2)	0.0250 (11)	
H6A	0.6424	0.2679	−0.0538	0.030*	
C7	0.4436 (3)	0.3220 (2)	−0.03952 (18)	0.0156 (9)	
C8	0.3745 (3)	0.3076 (3)	−0.09188 (19)	0.0226 (10)	
H8A	0.3503	0.2638	−0.0991	0.027*	
C9	0.3421 (4)	0.3579 (3)	−0.1327 (2)	0.0262 (11)	
H9A	0.2968	0.3477	−0.1673	0.031*	
C10	0.3765 (4)	0.4235 (3)	−0.1224 (2)	0.0291 (12)	
H10A	0.3531	0.4574	−0.1496	0.035*	
C11	0.4460 (4)	0.4384 (3)	−0.0714 (2)	0.0250 (11)	
H11A	0.4706	0.4822	−0.0649	0.030*	
C12	0.4791 (3)	0.3878 (2)	−0.02999 (19)	0.0193 (10)	
H12A	0.5252	0.3981	0.0043	0.023*	
C13	0.5760 (3)	0.2904 (2)	0.07033 (18)	0.0151 (9)	
H13A	0.6342	0.3028	0.0506	0.018*	
H13B	0.5500	0.3312	0.0865	0.018*	
C14	0.6133 (3)	0.2414 (2)	0.12239 (18)	0.0150 (9)	
H14A	0.6870	0.2470	0.1343	0.018*	
H14B	0.6011	0.1953	0.1084	0.018*	
C15	0.6075 (3)	0.3335 (2)	0.21966 (17)	0.0136 (9)	
C16	0.5496 (3)	0.3908 (2)	0.22665 (19)	0.0197 (10)	
H16A	0.4783	0.3895	0.2156	0.024*	
C17	0.5960 (4)	0.4502 (2)	0.2499 (2)	0.0218 (10)	
H17A	0.5562	0.4882	0.2543	0.026*	
C18	0.7018 (4)	0.4523 (2)	0.2664 (2)	0.0214 (10)	
H18A	0.7333	0.4920	0.2818	0.026*	
C19	0.7608 (3)	0.3960 (2)	0.26024 (19)	0.0218 (10)	
H19A	0.8319	0.3977	0.2718	0.026*	
C20	0.7143 (3)	0.3364 (2)	0.23677 (19)	0.0197 (10)	
H20A	0.7545	0.2985	0.2325	0.024*	
C21	0.6075 (3)	0.1921 (2)	0.24149 (18)	0.0135 (9)	
C22	0.5769 (3)	0.1893 (2)	0.29748 (19)	0.0202 (10)	
H22A	0.5294	0.2206	0.3074	0.024*	
C23	0.6159 (3)	0.1407 (2)	0.33913 (19)	0.0212 (10)	
H23A	0.5939	0.1394	0.3764	0.025*	
C24	0.6868 (4)	0.0947 (3)	0.3253 (2)	0.0311 (12)	
H24A	0.7121	0.0616	0.3528	0.037*	
C25	0.7202 (5)	0.0980 (3)	0.2704 (2)	0.0489 (18)	
H25A	0.7694	0.0675	0.2611	0.059*	
C26	0.6809 (4)	0.1466 (3)	0.2288 (2)	0.0369 (14)	
H26A	0.7043	0.1485	0.1919	0.044*	
C27	−0.0055 (3)	0.1639 (2)	−0.02139 (18)	0.0132 (8)	
C28	−0.0395 (3)	0.0993 (2)	−0.0397 (2)	0.0177 (9)	
H28A	−0.0661	0.0709	−0.0129	0.021*	
C29	−0.0341 (3)	0.0772 (2)	−0.0971 (2)	0.0188 (9)	
H29A	−0.0577	0.0343	−0.1089	0.023*	
C30	0.0063 (3)	0.1187 (2)	−0.13713 (19)	0.0157 (9)	
C31	0.0399 (3)	0.1832 (2)	−0.12005 (19)	0.0163 (9)	
H31A	0.0656	0.2117	−0.1471	0.020*	
C32	0.0347 (3)	0.2047 (2)	−0.06233 (19)	0.0169 (9)	
H32A	0.0587	0.2476	−0.0506	0.020*	
C33	0.0562 (4)	0.1309 (3)	−0.2346 (2)	0.0265 (11)	
H33A	0.0556	0.1056	−0.2709	0.040*	
H33B	0.1262	0.1408	−0.2175	0.040*	
H33C	0.0190	0.1723	−0.2433	0.040*	
C34	−0.0929 (3)	0.2752 (2)	0.05044 (18)	0.0131 (8)	
C35	−0.1244 (3)	0.3061 (2)	−0.00462 (19)	0.0174 (9)	
H35A	−0.1096	0.2856	−0.0394	0.021*	
C36	−0.1773 (3)	0.3669 (2)	−0.00867 (19)	0.0185 (10)	
H36A	−0.1975	0.3870	−0.0459	0.022*	
C37	−0.1999 (3)	0.3977 (2)	0.04264 (19)	0.0168 (9)	
C38	−0.1715 (3)	0.3668 (2)	0.09850 (19)	0.0168 (9)	
H38A	−0.1889	0.3865	0.1330	0.020*	
C39	−0.1173 (3)	0.3066 (2)	0.10191 (19)	0.0166 (9)	
H39A	−0.0968	0.2867	0.1391	0.020*	
C40	−0.2656 (4)	0.4963 (2)	−0.0106 (2)	0.0249 (11)	
H40A	−0.2926	0.5401	−0.0033	0.037*	
H40B	−0.3138	0.4728	−0.0397	0.037*	
H40C	−0.2012	0.5013	−0.0256	0.037*	
C41	−0.0892 (3)	0.1312 (2)	0.09426 (18)	0.0131 (8)	
C42	−0.0492 (3)	0.0949 (2)	0.14551 (19)	0.0177 (9)	
H42A	0.0181	0.1031	0.1638	0.021*	
C43	−0.1069 (3)	0.0473 (2)	0.1695 (2)	0.0184 (9)	
H43A	−0.0794	0.0245	0.2043	0.022*	
C44	−0.2068 (3)	0.0331 (2)	0.14173 (19)	0.0162 (9)	
C45	−0.2488 (3)	0.0692 (2)	0.09152 (19)	0.0186 (9)	
H45A	−0.3161	0.0607	0.0734	0.022*	
C46	−0.1902 (3)	0.1182 (2)	0.06823 (18)	0.0174 (9)	
H46A	−0.2191	0.1427	0.0347	0.021*	
C47	−0.3628 (3)	−0.0276 (3)	0.1468 (2)	0.0278 (11)	
H47A	−0.3879	−0.0643	0.1683	0.042*	
H47B	−0.3711	−0.0384	0.1048	0.042*	
H47C	−0.4012	0.0126	0.1524	0.042*	
C48	0.4032 (3)	0.1360 (2)	0.08991 (19)	0.0171 (9)	
C49	0.2750 (3)	0.1535 (2)	−0.0164 (2)	0.0191 (10)	
C50	0.2461 (3)	0.2880 (2)	0.01819 (18)	0.0161 (9)	
C51	0.3595 (3)	0.3359 (2)	0.12580 (19)	0.0164 (9)	
C52	0.3433 (3)	0.2911 (2)	0.24342 (19)	0.0154 (9)	
C53	0.3647 (3)	0.1630 (2)	0.20333 (18)	0.0132 (9)	
C54	0.1966 (3)	0.1227 (2)	0.11232 (19)	0.0167 (9)	
C55	0.1310 (3)	0.2102 (2)	0.1980 (2)	0.0189 (9)	
C56	0.1434 (3)	0.3135 (2)	0.11644 (19)	0.0167 (9)	
Cl1	0.10327 (11)	0.02867 (8)	0.30956 (6)	0.0430 (4)	
Cl2	0.00052 (16)	0.14019 (7)	0.35662 (7)	0.0564 (5)	
Cl3	−0.00018 (13)	0.01080 (9)	0.41315 (10)	0.0681 (6)	
C57	0.0695 (4)	0.0661 (3)	0.3742 (2)	0.0300 (12)	
H57A	0.1334	0.0779	0.4006	0.036*	

### Atomic displacement parameters (Å^2^)

**Table:**

	*U*^11^	*U*^22^	*U*^33^	*U*^12^	*U*^13^	*U*^23^
Ru1	0.00831 (15)	0.01602 (18)	0.01108 (15)	−0.00078 (13)	0.00201 (12)	−0.00159 (13)
Ru2	0.00786 (14)	0.01352 (17)	0.01036 (15)	−0.00059 (13)	0.00236 (11)	−0.00060 (13)
Ru3	0.00776 (15)	0.01449 (18)	0.01302 (16)	−0.00043 (13)	0.00287 (12)	−0.00031 (13)
As1	0.00888 (19)	0.0150 (2)	0.0132 (2)	−0.00037 (16)	0.00234 (16)	0.00067 (17)
P1	0.0102 (5)	0.0199 (6)	0.0097 (5)	−0.0008 (4)	0.0026 (4)	0.0000 (4)
P2	0.0093 (5)	0.0161 (6)	0.0102 (5)	−0.0001 (4)	0.0016 (4)	−0.0008 (4)
O1	0.0218 (17)	0.0224 (18)	0.0243 (17)	0.0062 (14)	0.0037 (14)	0.0004 (14)
O2	0.0264 (19)	0.039 (2)	0.037 (2)	0.0087 (16)	−0.0064 (16)	−0.0211 (18)
O3	0.0186 (16)	0.0217 (18)	0.0212 (16)	0.0022 (14)	0.0011 (13)	0.0018 (14)
O4	0.0225 (17)	0.0190 (17)	0.0229 (17)	−0.0034 (14)	−0.0012 (14)	0.0037 (14)
O5	0.0300 (19)	0.0285 (19)	0.0201 (17)	0.0002 (15)	0.0087 (14)	−0.0084 (15)
O6	0.0262 (18)	0.0181 (18)	0.0207 (16)	−0.0035 (14)	−0.0001 (14)	0.0019 (14)
O7	0.0188 (16)	0.0182 (18)	0.0310 (18)	−0.0009 (13)	0.0035 (14)	0.0014 (14)
O8	0.034 (2)	0.035 (2)	0.0195 (17)	−0.0052 (16)	0.0118 (15)	−0.0001 (15)
O9	0.0166 (16)	0.0200 (19)	0.042 (2)	0.0042 (14)	0.0095 (15)	−0.0027 (16)
O10	0.0163 (15)	0.0230 (17)	0.0185 (15)	−0.0035 (13)	0.0070 (13)	−0.0067 (13)
O11	0.0237 (17)	0.0217 (18)	0.0208 (16)	0.0103 (14)	0.0082 (13)	0.0040 (14)
O12	0.0116 (15)	0.0227 (18)	0.0271 (17)	−0.0014 (13)	0.0048 (13)	0.0060 (14)
C1	0.011 (2)	0.026 (3)	0.0108 (19)	0.0017 (18)	0.0022 (16)	−0.0015 (18)
C2	0.010 (2)	0.032 (3)	0.019 (2)	0.0001 (19)	0.0027 (17)	−0.008 (2)
C3	0.020 (2)	0.033 (3)	0.026 (3)	0.003 (2)	−0.001 (2)	−0.012 (2)
C4	0.016 (2)	0.047 (3)	0.018 (2)	0.008 (2)	0.0053 (19)	−0.011 (2)
C5	0.018 (2)	0.055 (4)	0.021 (2)	0.005 (2)	0.0088 (19)	0.000 (2)
C6	0.023 (2)	0.032 (3)	0.020 (2)	0.001 (2)	0.0025 (19)	0.000 (2)
C7	0.012 (2)	0.027 (3)	0.0095 (19)	0.0023 (18)	0.0073 (16)	0.0045 (17)
C8	0.018 (2)	0.036 (3)	0.014 (2)	−0.004 (2)	0.0049 (18)	0.003 (2)
C9	0.020 (2)	0.048 (3)	0.012 (2)	0.002 (2)	0.0048 (18)	0.007 (2)
C10	0.027 (3)	0.040 (3)	0.023 (2)	0.017 (2)	0.011 (2)	0.013 (2)
C11	0.032 (3)	0.023 (3)	0.023 (2)	0.006 (2)	0.014 (2)	0.005 (2)
C12	0.019 (2)	0.025 (3)	0.015 (2)	0.0044 (19)	0.0082 (18)	0.0017 (19)
C13	0.0114 (19)	0.021 (2)	0.0140 (19)	−0.0030 (17)	0.0044 (16)	0.0026 (18)
C14	0.0113 (19)	0.021 (2)	0.0127 (19)	−0.0003 (17)	0.0009 (16)	−0.0006 (17)
C15	0.014 (2)	0.018 (2)	0.0087 (18)	−0.0061 (17)	0.0017 (16)	−0.0009 (16)
C16	0.018 (2)	0.022 (2)	0.019 (2)	−0.0064 (19)	0.0013 (18)	0.0018 (19)
C17	0.024 (2)	0.018 (2)	0.023 (2)	−0.0043 (19)	0.0020 (19)	−0.0013 (19)
C18	0.023 (2)	0.023 (3)	0.019 (2)	−0.010 (2)	0.0051 (19)	−0.0025 (19)
C19	0.012 (2)	0.035 (3)	0.019 (2)	−0.010 (2)	0.0041 (18)	−0.002 (2)
C20	0.016 (2)	0.026 (3)	0.018 (2)	−0.0036 (19)	0.0050 (18)	−0.0011 (19)
C21	0.014 (2)	0.015 (2)	0.0119 (19)	−0.0012 (16)	0.0018 (16)	−0.0004 (16)
C22	0.015 (2)	0.029 (3)	0.018 (2)	0.0029 (19)	0.0063 (18)	−0.0003 (19)
C23	0.017 (2)	0.036 (3)	0.012 (2)	−0.001 (2)	0.0052 (17)	0.0026 (19)
C24	0.047 (3)	0.028 (3)	0.017 (2)	0.013 (2)	0.000 (2)	0.004 (2)
C25	0.073 (4)	0.055 (4)	0.021 (3)	0.049 (4)	0.012 (3)	0.004 (3)
C26	0.053 (4)	0.047 (4)	0.012 (2)	0.032 (3)	0.011 (2)	0.003 (2)
C27	0.0091 (19)	0.017 (2)	0.0137 (19)	0.0009 (16)	0.0021 (15)	−0.0001 (17)
C28	0.013 (2)	0.017 (2)	0.025 (2)	−0.0036 (17)	0.0081 (18)	0.0014 (19)
C29	0.016 (2)	0.015 (2)	0.027 (2)	−0.0031 (18)	0.0082 (18)	−0.0078 (19)
C30	0.0090 (19)	0.021 (2)	0.018 (2)	0.0044 (17)	0.0028 (16)	−0.0031 (18)
C31	0.012 (2)	0.021 (2)	0.017 (2)	−0.0001 (17)	0.0035 (16)	0.0046 (18)
C32	0.013 (2)	0.016 (2)	0.020 (2)	−0.0030 (17)	−0.0011 (17)	0.0002 (18)
C33	0.030 (3)	0.035 (3)	0.016 (2)	−0.008 (2)	0.009 (2)	−0.003 (2)
C34	0.0088 (19)	0.015 (2)	0.016 (2)	−0.0014 (16)	0.0039 (15)	−0.0017 (17)
C35	0.016 (2)	0.023 (2)	0.014 (2)	0.0028 (18)	0.0043 (17)	−0.0040 (18)
C36	0.021 (2)	0.020 (2)	0.013 (2)	0.0050 (19)	0.0017 (18)	0.0024 (18)
C37	0.014 (2)	0.014 (2)	0.022 (2)	0.0006 (17)	0.0013 (17)	0.0020 (18)
C38	0.015 (2)	0.021 (2)	0.016 (2)	0.0015 (18)	0.0067 (17)	−0.0018 (18)
C39	0.011 (2)	0.025 (2)	0.014 (2)	−0.0022 (17)	0.0038 (16)	0.0034 (18)
C40	0.032 (3)	0.024 (3)	0.021 (2)	0.010 (2)	0.012 (2)	0.007 (2)
C41	0.0095 (19)	0.018 (2)	0.014 (2)	−0.0001 (16)	0.0084 (16)	−0.0024 (17)
C42	0.014 (2)	0.020 (2)	0.018 (2)	0.0023 (18)	−0.0010 (17)	0.0021 (18)
C43	0.017 (2)	0.019 (2)	0.019 (2)	0.0028 (18)	0.0026 (18)	0.0022 (19)
C44	0.016 (2)	0.014 (2)	0.021 (2)	0.0002 (17)	0.0088 (17)	−0.0032 (18)
C45	0.012 (2)	0.023 (2)	0.021 (2)	−0.0016 (18)	0.0022 (17)	0.0000 (19)
C46	0.017 (2)	0.023 (2)	0.011 (2)	0.0025 (18)	0.0010 (17)	0.0016 (18)
C47	0.014 (2)	0.027 (3)	0.042 (3)	−0.002 (2)	0.006 (2)	0.009 (2)
C48	0.013 (2)	0.025 (3)	0.013 (2)	−0.0073 (18)	0.0011 (17)	−0.0018 (18)
C49	0.014 (2)	0.022 (2)	0.020 (2)	0.0030 (18)	−0.0014 (18)	−0.0025 (19)
C50	0.0107 (19)	0.024 (2)	0.014 (2)	−0.0072 (18)	0.0034 (16)	−0.0024 (19)
C51	0.0063 (19)	0.025 (3)	0.017 (2)	−0.0029 (17)	0.0010 (16)	−0.0033 (19)
C52	0.011 (2)	0.018 (2)	0.018 (2)	0.0008 (17)	0.0053 (16)	0.0014 (18)
C53	0.0087 (19)	0.017 (2)	0.014 (2)	0.0003 (16)	0.0028 (16)	−0.0058 (18)
C54	0.009 (2)	0.023 (3)	0.017 (2)	−0.0014 (17)	−0.0006 (16)	0.0041 (18)
C55	0.014 (2)	0.017 (2)	0.027 (2)	0.0002 (18)	0.0047 (18)	−0.003 (2)
C56	0.0084 (19)	0.024 (3)	0.019 (2)	−0.0026 (17)	0.0041 (16)	−0.0015 (19)
Cl1	0.0401 (8)	0.0504 (9)	0.0371 (7)	0.0130 (7)	0.0012 (6)	−0.0164 (7)
Cl2	0.1012 (14)	0.0258 (8)	0.0519 (9)	0.0151 (8)	0.0433 (10)	0.0064 (7)
Cl3	0.0499 (10)	0.0429 (10)	0.1242 (17)	0.0157 (8)	0.0542 (11)	0.0373 (10)
C57	0.027 (3)	0.035 (3)	0.030 (3)	−0.009 (2)	0.012 (2)	−0.005 (2)

### Geometric parameters (Å, °)

**Table:**

Ru1—C49	1.899 (4)	C15—C16	1.387 (6)
Ru1—C48	1.926 (5)	C15—C20	1.396 (6)
Ru1—C50	1.945 (5)	C16—C17	1.390 (6)
Ru1—P1	2.3291 (11)	C16—H16A	0.9300
Ru1—Ru2	2.8439 (4)	C17—C18	1.381 (6)
Ru1—Ru3	2.8694 (5)	C17—H17A	0.9300
Ru2—C52	1.886 (4)	C18—C19	1.376 (7)
Ru2—C51	1.922 (5)	C18—H18A	0.9300
Ru2—C53	1.932 (4)	C19—C20	1.396 (6)
Ru2—P2	2.3225 (11)	C19—H19A	0.9300
Ru2—Ru3	2.8221 (4)	C20—H20A	0.9300
Ru3—C55	1.875 (5)	C21—C26	1.380 (6)
Ru3—C54	1.924 (5)	C21—C22	1.383 (6)
Ru3—C56	1.935 (5)	C22—C23	1.387 (6)
Ru3—As1	2.4558 (5)	C22—H22A	0.9300
As1—C41	1.942 (4)	C23—C24	1.371 (7)
As1—C27	1.944 (4)	C23—H23A	0.9300
As1—C34	1.946 (4)	C24—C25	1.377 (7)
P1—C7	1.827 (4)	C24—H24A	0.9300
P1—C1	1.836 (5)	C25—C26	1.387 (7)
P1—C13	1.845 (4)	C25—H25A	0.9300
P2—C21	1.828 (4)	C26—H26A	0.9300
P2—C14	1.837 (4)	C27—C32	1.390 (6)
P2—C15	1.837 (4)	C27—C28	1.397 (6)
O1—C48	1.146 (5)	C28—C29	1.378 (6)
O2—C49	1.141 (5)	C28—H28A	0.9300
O3—C50	1.144 (5)	C29—C30	1.384 (6)
O4—C51	1.157 (5)	C29—H29A	0.9300
O5—C52	1.149 (5)	C30—C31	1.388 (6)
O6—C53	1.148 (5)	C31—C32	1.381 (6)
O7—C54	1.152 (5)	C31—H31A	0.9300
O8—C55	1.155 (5)	C32—H32A	0.9300
O9—C56	1.147 (5)	C33—H33A	0.9600
O10—C30	1.373 (5)	C33—H33B	0.9600
O10—C33	1.419 (5)	C33—H33C	0.9600
O11—C37	1.372 (5)	C34—C35	1.391 (6)
O11—C40	1.432 (5)	C34—C39	1.397 (6)
O12—C44	1.367 (5)	C35—C36	1.386 (6)
O12—C47	1.430 (5)	C35—H35A	0.9300
C1—C2	1.382 (6)	C36—C37	1.380 (6)
C1—C6	1.415 (6)	C36—H36A	0.9300
C2—C3	1.393 (6)	C37—C38	1.400 (6)
C2—H2A	0.9300	C38—C39	1.384 (6)
C3—C4	1.373 (7)	C38—H38A	0.9300
C3—H3A	0.9300	C39—H39A	0.9300
C4—C5	1.375 (8)	C40—H40A	0.9600
C4—H4A	0.9300	C40—H40B	0.9600
C5—C6	1.386 (7)	C40—H40C	0.9600
C5—H5A	0.9300	C41—C46	1.389 (6)
C6—H6A	0.9300	C41—C42	1.395 (6)
C7—C12	1.389 (6)	C42—C43	1.370 (6)
C7—C8	1.405 (6)	C42—H42A	0.9300
C8—C9	1.378 (7)	C43—C44	1.393 (6)
C8—H8A	0.9300	C43—H43A	0.9300
C9—C10	1.382 (7)	C44—C45	1.381 (6)
C9—H9A	0.9300	C45—C46	1.389 (6)
C10—C11	1.387 (7)	C45—H45A	0.9300
C10—H10A	0.9300	C46—H46A	0.9300
C11—C12	1.394 (6)	C47—H47A	0.9600
C11—H11A	0.9300	C47—H47B	0.9600
C12—H12A	0.9300	C47—H47C	0.9600
C13—C14	1.545 (6)	Cl1—C57	1.751 (5)
C13—H13A	0.9700	Cl2—C57	1.737 (6)
C13—H13B	0.9700	Cl3—C57	1.746 (5)
C14—H14A	0.9700	C57—H57A	0.9800
C14—H14B	0.9700		
			
C49—Ru1—C48	91.33 (18)	C17—C16—H16A	119.4
C49—Ru1—C50	94.22 (18)	C18—C17—C16	119.5 (5)
C48—Ru1—C50	171.82 (18)	C18—C17—H17A	120.2
C49—Ru1—P1	100.57 (14)	C16—C17—H17A	120.2
C48—Ru1—P1	93.20 (13)	C19—C18—C17	120.3 (4)
C50—Ru1—P1	91.67 (13)	C19—C18—H18A	119.9
C49—Ru1—Ru2	156.51 (14)	C17—C18—H18A	119.9
C48—Ru1—Ru2	75.81 (13)	C18—C19—C20	120.2 (4)
C50—Ru1—Ru2	96.90 (12)	C18—C19—H19A	119.9
P1—Ru1—Ru2	99.71 (3)	C20—C19—H19A	119.9
C49—Ru1—Ru3	104.31 (14)	C19—C20—C15	120.1 (4)
C48—Ru1—Ru3	97.61 (13)	C19—C20—H20A	119.9
C50—Ru1—Ru3	75.21 (12)	C15—C20—H20A	119.9
P1—Ru1—Ru3	152.54 (3)	C26—C21—C22	118.1 (4)
Ru2—Ru1—Ru3	59.199 (11)	C26—C21—P2	123.4 (3)
C52—Ru2—C51	96.26 (18)	C22—C21—P2	118.5 (3)
C52—Ru2—C53	90.92 (18)	C21—C22—C23	121.2 (4)
C51—Ru2—C53	170.33 (17)	C21—C22—H22A	119.4
C52—Ru2—P2	98.79 (13)	C23—C22—H22A	119.4
C51—Ru2—P2	93.15 (12)	C24—C23—C22	120.0 (4)
C53—Ru2—P2	92.13 (12)	C24—C23—H23A	120.0
C52—Ru2—Ru3	99.71 (12)	C22—C23—H23A	120.0
C51—Ru2—Ru3	91.50 (12)	C23—C24—C25	119.5 (5)
C53—Ru2—Ru3	80.89 (11)	C23—C24—H24A	120.3
P2—Ru2—Ru3	160.31 (3)	C25—C24—H24A	120.3
C52—Ru2—Ru1	158.34 (12)	C24—C25—C26	120.4 (5)
C51—Ru2—Ru1	76.13 (13)	C24—C25—H25A	119.8
C53—Ru2—Ru1	94.86 (12)	C26—C25—H25A	119.8
P2—Ru2—Ru1	101.83 (3)	C21—C26—C25	120.8 (5)
Ru3—Ru2—Ru1	60.850 (11)	C21—C26—H26A	119.6
C55—Ru3—C54	96.09 (19)	C25—C26—H26A	119.6
C55—Ru3—C56	93.41 (19)	C32—C27—C28	118.2 (4)
C54—Ru3—C56	170.41 (19)	C32—C27—As1	119.1 (3)
C55—Ru3—As1	100.39 (13)	C28—C27—As1	122.6 (3)
C54—Ru3—As1	88.34 (12)	C29—C28—C27	120.5 (4)
C56—Ru3—As1	91.27 (12)	C29—C28—H28A	119.7
C55—Ru3—Ru2	89.58 (13)	C27—C28—H28A	119.7
C54—Ru3—Ru2	94.72 (12)	C28—C29—C30	120.3 (4)
C56—Ru3—Ru2	83.98 (12)	C28—C29—H29A	119.9
As1—Ru3—Ru2	169.216 (19)	C30—C29—H29A	119.9
C55—Ru3—Ru1	145.84 (13)	O10—C30—C29	116.2 (4)
C54—Ru3—Ru1	73.39 (13)	O10—C30—C31	123.6 (4)
C56—Ru3—Ru1	97.89 (13)	C29—C30—C31	120.1 (4)
As1—Ru3—Ru1	111.410 (17)	C32—C31—C30	119.1 (4)
Ru2—Ru3—Ru1	59.951 (11)	C32—C31—H31A	120.4
C41—As1—C27	103.39 (17)	C30—C31—H31A	120.4
C41—As1—C34	101.75 (17)	C31—C32—C27	121.7 (4)
C27—As1—C34	103.68 (17)	C31—C32—H32A	119.2
C41—As1—Ru3	114.73 (12)	C27—C32—H32A	119.2
C27—As1—Ru3	117.48 (12)	O10—C33—H33A	109.5
C34—As1—Ru3	113.92 (12)	O10—C33—H33B	109.5
C7—P1—C1	102.4 (2)	H33A—C33—H33B	109.5
C7—P1—C13	102.9 (2)	O10—C33—H33C	109.5
C1—P1—C13	101.18 (19)	H33A—C33—H33C	109.5
C7—P1—Ru1	112.81 (14)	H33B—C33—H33C	109.5
C1—P1—Ru1	117.63 (15)	C35—C34—C39	118.2 (4)
C13—P1—Ru1	117.75 (14)	C35—C34—As1	123.1 (3)
C21—P2—C14	103.13 (19)	C39—C34—As1	118.3 (3)
C21—P2—C15	101.82 (18)	C36—C35—C34	121.3 (4)
C14—P2—C15	102.47 (19)	C36—C35—H35A	119.3
C21—P2—Ru2	114.83 (14)	C34—C35—H35A	119.3
C14—P2—Ru2	116.38 (13)	C37—C36—C35	119.8 (4)
C15—P2—Ru2	116.16 (14)	C37—C36—H36A	120.1
C30—O10—C33	118.5 (3)	C35—C36—H36A	120.1
C37—O11—C40	116.4 (3)	O11—C37—C36	124.9 (4)
C44—O12—C47	117.4 (3)	O11—C37—C38	115.1 (4)
C2—C1—C6	117.8 (4)	C36—C37—C38	120.0 (4)
C2—C1—P1	122.7 (3)	C39—C38—C37	119.4 (4)
C6—C1—P1	119.5 (4)	C39—C38—H38A	120.3
C1—C2—C3	121.2 (4)	C37—C38—H38A	120.3
C1—C2—H2A	119.4	C38—C39—C34	121.2 (4)
C3—C2—H2A	119.4	C38—C39—H39A	119.4
C4—C3—C2	120.2 (5)	C34—C39—H39A	119.4
C4—C3—H3A	119.9	O11—C40—H40A	109.5
C2—C3—H3A	119.9	O11—C40—H40B	109.5
C3—C4—C5	119.9 (5)	H40A—C40—H40B	109.5
C3—C4—H4A	120.1	O11—C40—H40C	109.5
C5—C4—H4A	120.1	H40A—C40—H40C	109.5
C4—C5—C6	120.7 (5)	H40B—C40—H40C	109.5
C4—C5—H5A	119.7	C46—C41—C42	118.0 (4)
C6—C5—H5A	119.7	C46—C41—As1	121.8 (3)
C5—C6—C1	120.2 (5)	C42—C41—As1	120.2 (3)
C5—C6—H6A	119.9	C43—C42—C41	121.4 (4)
C1—C6—H6A	119.9	C43—C42—H42A	119.3
C12—C7—C8	118.6 (4)	C41—C42—H42A	119.3
C12—C7—P1	124.0 (3)	C42—C43—C44	119.9 (4)
C8—C7—P1	117.3 (4)	C42—C43—H43A	120.0
C9—C8—C7	120.6 (5)	C44—C43—H43A	120.0
C9—C8—H8A	119.7	O12—C44—C45	125.5 (4)
C7—C8—H8A	119.7	O12—C44—C43	114.8 (4)
C8—C9—C10	120.5 (4)	C45—C44—C43	119.7 (4)
C8—C9—H9A	119.8	C44—C45—C46	119.8 (4)
C10—C9—H9A	119.8	C44—C45—H45A	120.1
C9—C10—C11	119.7 (4)	C46—C45—H45A	120.1
C9—C10—H10A	120.2	C45—C46—C41	121.0 (4)
C11—C10—H10A	120.2	C45—C46—H46A	119.5
C10—C11—C12	120.1 (5)	C41—C46—H46A	119.5
C10—C11—H11A	119.9	O12—C47—H47A	109.5
C12—C11—H11A	119.9	O12—C47—H47B	109.5
C7—C12—C11	120.5 (4)	H47A—C47—H47B	109.5
C7—C12—H12A	119.8	O12—C47—H47C	109.5
C11—C12—H12A	119.8	H47A—C47—H47C	109.5
C14—C13—P1	112.5 (3)	H47B—C47—H47C	109.5
C14—C13—H13A	109.1	O1—C48—Ru1	172.1 (4)
P1—C13—H13A	109.1	O2—C49—Ru1	174.1 (4)
C14—C13—H13B	109.1	O3—C50—Ru1	172.3 (4)
P1—C13—H13B	109.1	O4—C51—Ru2	173.3 (4)
H13A—C13—H13B	107.8	O5—C52—Ru2	179.4 (4)
C13—C14—P2	112.8 (3)	O6—C53—Ru2	175.5 (4)
C13—C14—H14A	109.0	O7—C54—Ru3	172.7 (4)
P2—C14—H14A	109.0	O8—C55—Ru3	175.9 (4)
C13—C14—H14B	109.0	O9—C56—Ru3	175.4 (4)
P2—C14—H14B	109.0	Cl2—C57—Cl3	110.5 (3)
H14A—C14—H14B	107.8	Cl2—C57—Cl1	110.9 (3)
C16—C15—C20	118.6 (4)	Cl3—C57—Cl1	111.8 (3)
C16—C15—P2	121.9 (3)	Cl2—C57—H57A	107.8
C20—C15—P2	119.5 (3)	Cl3—C57—H57A	107.8
C15—C16—C17	121.2 (4)	Cl1—C57—H57A	107.8
C15—C16—H16A	119.4		
			
C49—Ru1—Ru2—C52	−77.7 (5)	C7—P1—C1—C2	−133.3 (4)
C48—Ru1—Ru2—C52	−136.2 (4)	C13—P1—C1—C2	120.7 (4)
C50—Ru1—Ru2—C52	40.0 (4)	Ru1—P1—C1—C2	−9.1 (4)
P1—Ru1—Ru2—C52	132.9 (4)	C7—P1—C1—C6	46.2 (4)
Ru3—Ru1—Ru2—C52	−28.2 (4)	C13—P1—C1—C6	−59.8 (4)
C49—Ru1—Ru2—C51	−149.2 (4)	Ru1—P1—C1—C6	170.4 (3)
C48—Ru1—Ru2—C51	152.25 (18)	C6—C1—C2—C3	1.0 (6)
C50—Ru1—Ru2—C51	−31.51 (17)	P1—C1—C2—C3	−179.5 (3)
P1—Ru1—Ru2—C51	61.39 (13)	C1—C2—C3—C4	−1.1 (7)
Ru3—Ru1—Ru2—C51	−99.70 (12)	C2—C3—C4—C5	0.4 (7)
C49—Ru1—Ru2—C53	27.2 (3)	C3—C4—C5—C6	0.4 (7)
C48—Ru1—Ru2—C53	−31.31 (18)	C4—C5—C6—C1	−0.4 (7)
C50—Ru1—Ru2—C53	144.93 (17)	C2—C1—C6—C5	−0.2 (6)
P1—Ru1—Ru2—C53	−122.17 (12)	P1—C1—C6—C5	−179.8 (3)
Ru3—Ru1—Ru2—C53	76.74 (12)	C1—P1—C7—C12	−115.5 (4)
C49—Ru1—Ru2—P2	120.5 (3)	C13—P1—C7—C12	−10.8 (4)
C48—Ru1—Ru2—P2	61.90 (13)	Ru1—P1—C7—C12	117.1 (4)
C50—Ru1—Ru2—P2	−121.86 (12)	C1—P1—C7—C8	69.0 (4)
P1—Ru1—Ru2—P2	−28.97 (4)	C13—P1—C7—C8	173.7 (3)
Ru3—Ru1—Ru2—P2	169.94 (3)	Ru1—P1—C7—C8	−58.3 (4)
C49—Ru1—Ru2—Ru3	−49.5 (3)	C12—C7—C8—C9	−0.4 (7)
C48—Ru1—Ru2—Ru3	−108.05 (13)	P1—C7—C8—C9	175.3 (4)
C50—Ru1—Ru2—Ru3	68.19 (12)	C7—C8—C9—C10	−0.5 (7)
P1—Ru1—Ru2—Ru3	161.09 (3)	C8—C9—C10—C11	1.5 (7)
C52—Ru2—Ru3—C55	−26.39 (19)	C9—C10—C11—C12	−1.5 (7)
C51—Ru2—Ru3—C55	−123.00 (19)	C8—C7—C12—C11	0.4 (6)
C53—Ru2—Ru3—C55	62.98 (18)	P1—C7—C12—C11	−175.0 (3)
P2—Ru2—Ru3—C55	133.32 (16)	C10—C11—C12—C7	0.6 (7)
Ru1—Ru2—Ru3—C55	163.80 (14)	C7—P1—C13—C14	178.7 (3)
C52—Ru2—Ru3—C54	−122.47 (19)	C1—P1—C13—C14	−75.7 (3)
C51—Ru2—Ru3—C54	140.92 (19)	Ru1—P1—C13—C14	54.0 (3)
C53—Ru2—Ru3—C54	−33.09 (18)	P1—C13—C14—P2	−95.4 (3)
P2—Ru2—Ru3—C54	37.25 (16)	C21—P2—C14—C13	−179.3 (3)
Ru1—Ru2—Ru3—C54	67.73 (13)	C15—P2—C14—C13	−73.8 (3)
C52—Ru2—Ru3—C56	67.08 (18)	Ru2—P2—C14—C13	54.1 (3)
C51—Ru2—Ru3—C56	−29.53 (18)	C21—P2—C15—C16	−132.4 (4)
C53—Ru2—Ru3—C56	156.45 (18)	C14—P2—C15—C16	121.1 (4)
P2—Ru2—Ru3—C56	−133.21 (16)	Ru2—P2—C15—C16	−6.9 (4)
Ru1—Ru2—Ru3—C56	−102.73 (13)	C21—P2—C15—C20	48.3 (4)
C52—Ru2—Ru3—As1	131.37 (17)	C14—P2—C15—C20	−58.2 (4)
C51—Ru2—Ru3—As1	34.76 (17)	Ru2—P2—C15—C20	173.8 (3)
C53—Ru2—Ru3—As1	−139.26 (16)	C20—C15—C16—C17	0.3 (6)
P2—Ru2—Ru3—As1	−68.92 (15)	P2—C15—C16—C17	−179.0 (3)
Ru1—Ru2—Ru3—As1	−38.44 (10)	C15—C16—C17—C18	0.0 (7)
C52—Ru2—Ru3—Ru1	169.81 (13)	C16—C17—C18—C19	−0.4 (7)
C51—Ru2—Ru3—Ru1	73.20 (13)	C17—C18—C19—C20	0.6 (7)
C53—Ru2—Ru3—Ru1	−100.82 (12)	C18—C19—C20—C15	−0.3 (7)
P2—Ru2—Ru3—Ru1	−30.47 (9)	C16—C15—C20—C19	−0.1 (6)
C49—Ru1—Ru3—C55	132.0 (3)	P2—C15—C20—C19	179.1 (3)
C48—Ru1—Ru3—C55	38.6 (3)	C14—P2—C21—C26	−2.0 (5)
C50—Ru1—Ru3—C55	−137.4 (3)	C15—P2—C21—C26	−108.0 (4)
P1—Ru1—Ru3—C55	−73.6 (3)	Ru2—P2—C21—C26	125.6 (4)
Ru2—Ru1—Ru3—C55	−29.8 (2)	C14—P2—C21—C22	178.7 (3)
C49—Ru1—Ru3—C54	56.01 (18)	C15—P2—C21—C22	72.7 (4)
C48—Ru1—Ru3—C54	−37.34 (17)	Ru2—P2—C21—C22	−53.7 (4)
C50—Ru1—Ru3—C54	146.65 (17)	C26—C21—C22—C23	−2.3 (7)
P1—Ru1—Ru3—C54	−149.61 (14)	P2—C21—C22—C23	177.1 (3)
Ru2—Ru1—Ru3—C54	−105.77 (12)	C21—C22—C23—C24	0.6 (7)
C49—Ru1—Ru3—C56	−119.90 (18)	C22—C23—C24—C25	1.2 (8)
C48—Ru1—Ru3—C56	146.75 (17)	C23—C24—C25—C26	−1.4 (10)
C50—Ru1—Ru3—C56	−29.26 (17)	C22—C21—C26—C25	2.1 (8)
P1—Ru1—Ru3—C56	34.48 (14)	P2—C21—C26—C25	−177.2 (5)
Ru2—Ru1—Ru3—C56	78.32 (12)	C24—C25—C26—C21	−0.4 (10)
C49—Ru1—Ru3—As1	−25.40 (14)	C41—As1—C27—C32	169.6 (3)
C48—Ru1—Ru3—As1	−118.75 (12)	C34—As1—C27—C32	63.8 (3)
C50—Ru1—Ru3—As1	65.24 (12)	Ru3—As1—C27—C32	−62.9 (3)
P1—Ru1—Ru3—As1	128.98 (6)	C41—As1—C27—C28	−14.7 (4)
Ru2—Ru1—Ru3—As1	172.82 (2)	C34—As1—C27—C28	−120.5 (3)
C49—Ru1—Ru3—Ru2	161.77 (14)	Ru3—As1—C27—C28	112.8 (3)
C48—Ru1—Ru3—Ru2	68.43 (12)	C32—C27—C28—C29	−0.7 (6)
C50—Ru1—Ru3—Ru2	−107.58 (12)	As1—C27—C28—C29	−176.4 (3)
P1—Ru1—Ru3—Ru2	−43.85 (6)	C27—C28—C29—C30	0.7 (6)
C55—Ru3—As1—C41	−35.50 (19)	C33—O10—C30—C29	−175.5 (4)
C54—Ru3—As1—C41	60.39 (19)	C33—O10—C30—C31	5.6 (6)
C56—Ru3—As1—C41	−129.19 (19)	C28—C29—C30—O10	179.9 (4)
Ru2—Ru3—As1—C41	167.14 (16)	C28—C29—C30—C31	−1.2 (6)
Ru1—Ru3—As1—C41	131.82 (13)	O10—C30—C31—C32	−179.6 (4)
C55—Ru3—As1—C27	−157.3 (2)	C29—C30—C31—C32	1.5 (6)
C54—Ru3—As1—C27	−61.41 (19)	C30—C31—C32—C27	−1.5 (6)
C56—Ru3—As1—C27	109.00 (19)	C28—C27—C32—C31	1.0 (6)
Ru2—Ru3—As1—C27	45.33 (18)	As1—C27—C32—C31	176.9 (3)
Ru1—Ru3—As1—C27	10.02 (14)	C41—As1—C34—C35	−121.1 (4)
C55—Ru3—As1—C34	81.22 (19)	C27—As1—C34—C35	−14.0 (4)
C54—Ru3—As1—C34	177.11 (19)	Ru3—As1—C34—C35	114.8 (3)
C56—Ru3—As1—C34	−12.48 (19)	C41—As1—C34—C39	66.1 (3)
Ru2—Ru3—As1—C34	−76.15 (17)	C27—As1—C34—C39	173.2 (3)
Ru1—Ru3—As1—C34	−111.46 (14)	Ru3—As1—C34—C39	−58.0 (3)
C49—Ru1—P1—C7	79.4 (2)	C39—C34—C35—C36	0.9 (6)
C48—Ru1—P1—C7	171.4 (2)	As1—C34—C35—C36	−171.9 (3)
C50—Ru1—P1—C7	−15.2 (2)	C34—C35—C36—C37	−0.2 (7)
Ru2—Ru1—P1—C7	−112.51 (16)	C40—O11—C37—C36	−8.9 (6)
Ru3—Ru1—P1—C7	−75.37 (17)	C40—O11—C37—C38	170.9 (4)
C49—Ru1—P1—C1	−39.4 (2)	C35—C36—C37—O11	178.3 (4)
C48—Ru1—P1—C1	52.53 (19)	C35—C36—C37—C38	−1.5 (7)
C50—Ru1—P1—C1	−134.04 (19)	O11—C37—C38—C39	−177.3 (4)
Ru2—Ru1—P1—C1	128.67 (15)	C36—C37—C38—C39	2.4 (6)
Ru3—Ru1—P1—C1	165.80 (14)	C37—C38—C39—C34	−1.8 (6)
C49—Ru1—P1—C13	−160.9 (2)	C35—C34—C39—C38	0.1 (6)
C48—Ru1—P1—C13	−69.0 (2)	As1—C34—C39—C38	173.3 (3)
C50—Ru1—P1—C13	104.4 (2)	C27—As1—C41—C46	−56.4 (4)
Ru2—Ru1—P1—C13	7.16 (17)	C34—As1—C41—C46	50.9 (4)
Ru3—Ru1—P1—C13	44.29 (19)	Ru3—As1—C41—C46	174.4 (3)
C52—Ru2—P2—C21	73.6 (2)	C27—As1—C41—C42	122.2 (4)
C51—Ru2—P2—C21	170.4 (2)	C34—As1—C41—C42	−130.5 (3)
C53—Ru2—P2—C21	−17.69 (19)	Ru3—As1—C41—C42	−7.0 (4)
Ru3—Ru2—P2—C21	−86.20 (18)	C46—C41—C42—C43	0.5 (7)
Ru1—Ru2—P2—C21	−113.11 (15)	As1—C41—C42—C43	−178.1 (3)
C52—Ru2—P2—C14	−165.8 (2)	C41—C42—C43—C44	1.7 (7)
C51—Ru2—P2—C14	−69.0 (2)	C47—O12—C44—C45	−6.5 (6)
C53—Ru2—P2—C14	102.9 (2)	C47—O12—C44—C43	172.5 (4)
Ru3—Ru2—P2—C14	34.4 (2)	C42—C43—C44—O12	178.2 (4)
Ru1—Ru2—P2—C14	7.48 (16)	C42—C43—C44—C45	−2.7 (7)
C52—Ru2—P2—C15	−45.0 (2)	O12—C44—C45—C46	−179.5 (4)
C51—Ru2—P2—C15	51.8 (2)	C43—C44—C45—C46	1.5 (7)
C53—Ru2—P2—C15	−136.30 (19)	C44—C45—C46—C41	0.7 (7)
Ru3—Ru2—P2—C15	155.19 (15)	C42—C41—C46—C45	−1.7 (6)
Ru1—Ru2—P2—C15	128.28 (15)	As1—C41—C46—C45	176.8 (3)

### Hydrogen-bond geometry (Å, °)

**Table:**

Cg1, Cg2 and Cg3 are the centroids of the C34–C39, C7–C12 and C1–C6 benzene rings, respectively.

**Table:**

*D*—H···*A*	*D*—H	H···*A*	*D*···*A*	*D*—H···*A*
C19—H19A···O10^i^	0.93	2.34	3.269 (5)	176
C43—H43A···O10^ii^	0.93	2.52	3.065 (5)	118
C47—H47C···O1^iii^	0.96	2.56	3.400 (6)	146
C13—H13A···Cg1^iv^	0.97	2.96	3.856 (4)	155
C23—H23A···Cg2^v^	0.93	2.73	3.467 (4)	137
C46—H46A···Cg3^iii^	0.93	2.83	3.623 (4)	145

Symmetry codes: (i) *x*+1, −*y*+1/2, *z*+1/2; (ii) −*x*, −*y*, −*z*; (iii) *x*−1, *y*, *z*; (iv) *x*+1, *y*, *z*; (v) *x*, −*y*−1/2, *z*−1/2.
